# Malaria in pregnancy: the difficulties in measuring birthweight

**DOI:** 10.1111/j.1471-0528.2010.02880.x

**Published:** 2011-02-18

**Authors:** MJ Rijken, JA Rijken, AT Papageorghiou, SH Kennedy, GHA Visser, F Nosten, R McGready

**Affiliations:** a Shoklo Malaria Research UnitMae Sot, Tak, Thailand; b Department of Obstetrics, University Medical Centre Utrecht Utrechtthe Netherlands; c Nuffield Department of Obstetrics and Gynaecology, John Radcliffe HospitalOxford, UK; d Centre for Clinical Vaccinology and Tropical Medicine, Nuffield Department of Clinical Medicine, John Radcliffe Hospital, University of OxfordOxford, UK; e Faculty of Tropical Medicine, Mahidol University BangkokThailand

**Keywords:** Birthweight, gestational age, malaria, pregnancy

## Abstract

Recommendations for interventions to control malaria in pregnancy are often based on studies using birthweight as the primary endpoint. Differences in birthweight may be attributable partly to methodological difficulties. We performed a structured search of the literature using ‘malaria’, ‘pregnancy’ and ‘birth weight’ as search terms. Of the clinical trials reporting birthweight, only 33% (14/43) gave information about the timing of the measurement and details on the scales used. Seventy seven per cent explained how gestational age was estimated. We propose a standardised method for the measurement and reporting of birthweight in future studies.

## Introduction

Malaria in pregnancy has a major impact on the health of the mother and fetus. In endemic areas, malaria is estimated to be responsible for 20% of low-birthweight (LBW) infants, the greatest single risk factor for infant mortality.^1^–^3^ However, malaria can cause both intrauterine growth restriction (IUGR), related to the sequestration of malaria parasites in the placenta, and preterm labour (PTL), which is associated with symptomatic maternal illness in the third trimester. IUGR and PTL can be distinguished only if the gestational age is known with some accuracy.^4^–^6^ This can be difficult in resource-poor settings. Many published clinical trials on maternal malaria have recruited women at the time of delivery, and birthweight (but not IUGR or PTL) has been the major endpoint. Different types of intervention may have different impacts on IUGR and PTL.

In this article, we review the methods used to obtain and report birthweight in studies of malaria during pregnancy. We also propose a systematic method for the reporting of birth outcomes, with an emphasis on studies in resource-poor settings.

## Methods

A Medline (PubMed) search was performed with the search terms ‘malaria’ AND ‘pregnancy’ AND ‘birth weight’ using a combination of MeSH headings and keywords. The search was not designed to identify all studies on malaria in pregnancy, but to analyse those that included birthweight and malaria as outcomes. Only trials that specifically used birthweight as the main outcome were included.

The search was limited to humans, clinical trials, randomised controlled trials (RCTs), case reports and English language articles from 1 January 1966 to 23 July 2009. Full articles of all citations resulting from this search were obtained. We scrutinised all articles for details of the methodology used to obtain birthweight. Two investigators independently performed eligibility assessment and, if disagreements were not resolved by consensus, a decision was made by a third author. Data from the included studies were extracted and entered onto an Excel spreadsheet for collation and analysis. Information on the type of scales, precision of scales, scale calibration, day of weight, inclusion criteria for birthweight analysis, proportion of pregnant women enrolled, proportion of infants weighed and studies reporting significant difference in birthweight were extracted. When available, the method of gestational age estimation and the potential confounders of birthweight measurements were also extracted.

## Results

Sixty-three publications were identified. Three of these reports were excluded because one was a review article^7^ and two did not report birthweight.^8^,^9^ There were 43 different trials and three case reports described in the remaining articles. The case reports did not contain sufficient details on the methodology used to measure birthweight and were excluded.^10^–^12^ Forty-three studies described in 59 publications were reviewed (Table S1, see Supporting information).^13^–^72^ Most (56%, 24/43) were studies on the prevention of malaria by intermittent preventive treatment (IPTp) or chemoprophylaxis in the African subcontinent (Table S1).

### Weighing scales: model and accuracy

Different types of weighing scales were described, varying from spring scales used in field situations to very precise digital scales in referral hospitals. Only 44% (19/43) of the articles reported the type of scale (Table S2, see Supporting information). Calibration of scales with standard weights was reported in three studies.^46^,^55^,^68^ Scale precision varied from 1 to 100 g (Table S2).

In resource-limited, tropical, humid or dusty field conditions, electronic scales can break^56^ or trained midwives can be too busy to measure all babies;^15^ however, only two research teams reported such events. One study in Kenya confirmed birthweight measurement by a double reading, and used the mean value for data analysis.^68^

Table S2 shows the variation in the printed format of birthweight. Most articles provided the mean ± standard deviation (SD) birthweight in grams; those that reported a significant difference between groups (44% [19/43]) are highlighted in bold in the table. One study reported a significant difference of 80 g between two groups, although ‘birthweight at the two hospitals was recorded to the nearest 100 g’.^59^ Three articles only reported the proportions of LBW, but not the actual mean birthweight.^26^,^55^,^66^ The average percentage of newborns that were included for birthweight analysis was 71% (range, 33–100%). In 58% (11/19) of the studies that reported a significant difference in birthweight, the result was based on the analysis of <80% of the participants.

### Date of weighing the infant

Delay in the weighing of the infant has an effect on the reported result.^73^,^74^ Seventy per cent (30/43) of the publications reported the time interval between the delivery and measurement of birthweight, and 35% (15/43) included babies weighed within 24 hours of birth (Table S2). The percentage of babies that were included for birthweight analysis is shown in Table S2. Some studies had a low proportion (14%^53^ or 15%^68^) of infants weighed within 24 hours. Eight publications adjusted for the day of weight using one of two formulae.^35^,^75^ However, the actual formulae were not provided, and different articles derived different percentage adjustments despite referencing the same formula. For example, some authors adjusted the weights by 1% for weight measured on day 4,^33^ and others, using the same formula,^75^ used a 3% correction,^15^ whereas a 5% adjustment was made using the formula described by Greenwood et al.^35^

### Inclusion in birthweight analysis

Most of the included studies confined their analysis of birthweight to liveborn singletons (Table S2). Three publications included multiple deliveries (twins or triplets).^54^,^59^,^70^ Three teams specifically reported that twins and congenital abnormalities were excluded from further analysis.^41^,^43^,^72^ The remaining publications did not mention whether twins, stillborn infants or those with congenital abnormalities were included in the analysis. Congenital abnormalities were reported in 15 publications.^13^,^14^,^17^,^22^,^23^,^29^,^43^,^45^,^46^,^50^,^54^,^60^–^62^,^72^ Eight studies (18%) reported the length of the infant at birth and, of these, four reported IUGR.^41^,^50^,^64^,^72^

### Method of gestational age estimation

**Figure 1:**
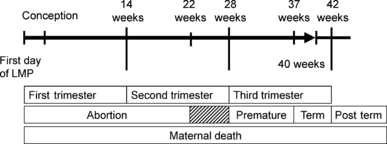
Definitions of pregnancy partitions. Maternal mortality can occur whilst pregnant or up to 42 days after termination of pregnancy.

Gestational age is defined as the time elapsed from the first day of the last menstrual period (LMP), if known, to the day of delivery.^76^ Gestational age can then be divided into blocks depending largely on neonatal viability (see Figure 1). The World Health Organization (WHO) uses a 22-week threshold to define miscarriage, but different definitions continue to be used. In the articles reviewed, the method of estimating gestational age was described in 77% (33/43) of the publications. Table 1 shows that the symphysis–fundal height (SFH) was the most commonly used method.

**Table 1:** Reporting of estimation of gestational age during pregnancy or in the postpartum period

Postnatal dating method	Pregnancy dating					Total
		
	None	LMP	SFH	LMP/SFH	US	
No postnatal test	10^19^,^20^,^23^,^27^,^35^,^47^,^49^,^58^,^59^,^69^	3^16^,^22^,^70^	8^28^,^32^,^33^,^40^,^51^,^53^,^55^,^60^	3^15^,^46^,^63^	4*^13^,^14^,^26^,^62^	28
Ballard	1^31^	0	1^45^	2^41^,^67^	0	4
Capurro	0	1^66^	0	0	0	1
Dubowitz	1^17^	1^64^	4^24^,^54^,^61^,^72^	1**^38^	1^50^	8
Lubchenko	1***^29^	0	0	0	0	1
Other	0	0	1****^56^	0	0	1
Total	13	5	14	6	5	43

LMP, last menstrual period; SFH, symphysis–fundal height; US, ultrasound.

* Ultrasound-derived gestational age assessment was used when menstrual dates were unknown (31%) or when the measurement of fetal size was more than two standard deviations above or below the mean for gestation calculated from the menstrual history (22%).^26^ Only women who could afford to pay had an ultrasound dating scan in Benin.^62^

** If the discrepancy between the LMP- and Dubowitz-derived gestational age was more than 14 days, the Dubowitz score was used.^38^

*** The gestational age was determined using the Ballard score. Anthropometric parameters and gestational age were used to classify the babies using a Lubchenko chart as preterm, term and postdate.^29^,^30^

**** Midwives and Mother and Child Health aids recorded newborns as being full term or premature using personal experiences and based on the indicators for rapid assessment of maturity.^56^

#### SFH

This measurement enables the gestational age to be calculated, but the formulae differ between populations.^72^,^77^,^78^ Several publications report SFH to be inaccurate^34^,^43^,^68^; however, it was the most common single method used to describe gestational age in this review (Table 1).^24^,^28^,^32^,^34^,^40^,^45^,^51^,^52^,^54^–^56^,^60^,^61^,^72^

#### LMP

The use of LMP was reported to be inaccurate in studies in which another method of gestational age estimation was available.^26^,^41^,^43^,^64^,^65^ Examples of such inaccuracies were because the women could not recall their LMP,^26^,^50^ or there was no menstruation between two consecutive pregnancies.^43^ One study reported 22% of pregnancies in which the ultrasound measurement of fetal size was more than two SDs above or below the mean for gestation calculated from the menstrual history.^26^ In another study, estimation of gestational age was based on the identification of ‘quickening’, which was interpreted as an indication that the gestational age was more than 18 weeks.^42^ A combination of LMP and SFH was used to estimate gestational age in six studies.^15^,^38^,^41^,^46^,^63^,^67^ One study that compared postnatal tests and LMP/SFH reported, ‘an unacceptably high number of women had pregnancies of more than 44 weeks, which makes the usefulness of LMP for assessment of gestational age doubtful’ and ‘gestational age derived from SFH is not reliable, with a range of 20–50 weeks’.^43^

#### Newborn gestational age assessment

The number of physical and neuromuscular maturity criteria examined in standardised neonatal scoring systems is critical to the accuracy of the test.^79^ However, a number of modifications to the methods of Ballard^43^ and Dubowitz^80^,^81^ have been used in resource-poor settings. Newborn tests have been reported to be less accurate when performed after 12–20 hours of age,^22^ and to be time consuming.^56^

#### Ultrasound gestational age assessment

Ultrasound measurement to estimate gestational age was reported from Kenya,^26^ Sudan^13^ and Thailand.^14^,^50^ In Benin, ultrasound scans were limited to patients who could afford to pay, but it was not reported how many women were scanned.^62^

### Confounding factors

Several factors were reported to have an impact on birthweight, including maternal smoking (*n* = 2),^66^,^72^ high blood pressure or pre-eclampsia (*n* = 9),^17^,^23^,^26^,^32^,^60^–^62^,^66^,^72^ maternal infections (e.g. chorioamnionitis) as risk factors for premature labour (*n* = 3),^29^,^45^,^61^ parity, height and nutritional status of the mother, number of antenatal clinic visits, rainy season and sex of the baby.^17^,^23^,^24^,^29^,^45^,^61^,^63^–^66^,^72^ The sex of the newborn was reported in 40% (17/43) of studies. A few authors make reference to the problem of ‘infants that were not weighed’, attributed to highly mobile rural populations and large numbers of home deliveries resulting in missing delivery information.^34^,^55^,^69^ These missing data may introduce bias, and one study showed that the loss of contact with subjects during follow-up was more prevalent in the control group than in the treated group.^20^

## Discussion

Considerable effort by researchers and the pregnant women themselves has been devoted to determine the impact of antimalarial interventions on birthweight, often under difficult conditions. Important information regarding the methodology and reporting of birth outcome data is often missing or inaccurately reported. Journal space restrictions may not allow authors to describe completely what they actually did and this is a potential limitation of our review. This article does not question the association between malaria and birthweight reduction, but highlights that the conclusions drawn about the effects of interventions based on differences in birthweight could partly be explained by inaccuracy in measurement methods.

### Gestational age estimation

When designing and conducting perinatal research studies, careful selection of the method of gestational age estimation is necessary, as findings can differ considerably according to the method.^82^ When differences in birthweight are found, a bias caused by the selection of a particular method must be considered as an alternative explanation for any association found.^82^

An error in gestational age estimation of even 1 week has major implications on birthweight. The weight gain of a fetus in the late third trimester can be as much as 250 g per week:^83^ this value is similar to the reduction in weight attributable to malaria.^1^–^3^ Ideally, gestational age should be estimated by fetal crown–rump length (CRL) or early second-trimester ultrasound, which is the standard in resource-rich countries^84^–^86^ and is becoming available in developing countries.^87^ When no reliable LMP, SFH or ultrasound measurements are available, postnatal examination of the newborn, with clinical scoring for external and/or neurological characteristics, can be used. These methods can be performed by locally trained paramedical health workers or nurses.^43^,^88^ The Dubowitz^80^ examination for the estimation of gestational age is recommended from 6 to 72 hours of life, which can make it difficult to include home births.

### Weighing scales and reporting of birthweight data

All that is needed to measure newborn weight is a scale. As a result, birthweight is frequently used as the only item to describe birth outcome. To describe the type of growth restriction caused by malaria, additional parameters, such as gestational age, newborn length and/or head circumference measurements, are required.

The accuracy of the equipment used to measure birthweight is paramount.^89^ It is preferable to use scales that have been registered for medical use, and the name, model and accuracy should be reported, especially if newborns are weighed at home. In the articles included here, none compared birthweight of home- and hospital-delivered babies, but this has the potential for large differences in measurements. Although some studies reported weight to the nearest gram,^29^,^31^,^54^,^64^ the reported accuracy by the manufacturer is usually of the order of 10 g, even though some digital scales may provide readouts to the nearest gram.

Research teams should be adequately trained in obtaining and reporting measurements. Ideally, research measurements should be taken by two different trained observers who are blind to the results obtained by the other, with measurements repeated that exceed preset maximum allowable differences.^90^ A standard method of calibrating scales should also take place at least once a week. With a sufficient sample size of newborns, birthweight is a normally distributed continuous variable, so that the presentation of such data would be expected to include the mean ± SD, as well as the minimum and maximum (range).

#### Date of weight

Normal birthweight reduction can be as much as 10% by day 3,^73^,^74^ and, in a 3000-g baby, this would result in a weight reduction of 300 g; this is within the order of magnitude of the effect described for malaria in pregnancy. Consequently, the day the newborn is weighed has important implications for research, particularly as a large proportion of women in resource-poor settings deliver at home, and delays in the recording of birthweight are expected. Ideally, birthweight should be obtained within 24 hours of birth or, if taken after 24 hours, a correction formula could be applied. However, blanket correction factors do not account for the differences in postnatal weight loss as a result of birthweight categories,^91^ gravidity/parity,^92^ race, asphyxia^92^ and age at the initiation of breastfeeding.^92^ In the context of RCTs, the proportion of infants weighed on different days should not be significantly different between the groups.

#### Birthweight analysis

The inclusion of multiple pregnancies, stillbirths or infants with congenital abnormalities will have an impact on birthweight analysis. Although such pregnancies need to be reported, they should not be included in any analysis of birthweight. Whether minor congenital abnormalities have an impact on birthweight is debatable but, for the sake of consistency, they should probably be excluded from any birthweight analysis in the context of clinical trials. Nevertheless, it is important to highlight that congenital abnormalities will not be reliably reported without an adequate examination of the newborn by a trained observer and a standardised method of classifying abnormalities. WHO has established a pregnancy registry (http://apps.who.int/tdr/svc/grants/calls/call-contributions) that will include the prevalence of birth defects in Asia, Africa and Latin-America, and classification using International Classification of Diseases (ICD10).

## Confounders

Most potential factors confounding birthweight can be detected by adequate antenatal and intrapartum care. Inclusion of gender in the analysis could be used as an internal validity check, as female newborns are lighter than males.^93^,^94^ Although clinical trials often exclude women with known medical or obstetric problems, some could arise after inclusion and should be controlled for, for example, hypertension (eclampsia, pre-eclampsia, pregnancy-induced hypertension), infections (pyelonephritis, sexually transmitted infections, local area-specific infections, e.g. typhoid, scrub typhus), obstetric problems, such as preterm labour, and whether there is a recognised trigger, for example, symptomatic malaria or pyelonephritis. RCTs could minimise the effect of potential confounders, but they should be included in any (multivariate) analysis.

Birthweight may not necessarily be the best method to evaluate the efficacy of interventions against malaria in pregnancy. The study of IUGR and the type of growth restriction (symmetrical or asymmetrical) requires additional parameters, including gestational age, newborn length and/or head circumference measurements.

## Conclusions

Differences in birthweight are often used to compare the efficacy of interventions aimed to reduce the impact of malaria during pregnancy.^95^–^98^ Such differences can clearly be affected by inaccuracies in measurement methods, and confounders such as those affecting gestational age or time of weighing. The reporting of birthweight and gestational age in maternal malaria studies can be improved. Simple methodological guidelines for reporting birth outcome, with an emphasis on studies of malaria in pregnancy, are provided (Table 2).

**Table 2:** Recommendations

**Birthweight**
•Measure birthweight on all newborns (alive or stillborn) as soon as possible after birth, preferably before 24 hours
•Only liveborn singletons without congenital abnormalities should be included in birthweight analysis
•Report the actual number of newborn babies included in the birthweight analysis
•Report the time interval (in days) between birth and the measurement of birthweight
•Certified medical scales should be used
•Report the name, model and accuracy of the weight scale and state whether the scale was sufficiently sensitive to detect any difference identified. Scales should be calibrated on a weekly basis
•Head circumference and length of the newborn should be measured
•Regular standardisation sessions and quality control checks of the measurers are required^90^
•The sex of the baby should be recorded
**Gestational age assessment**
•Report the method of estimating gestational age
•A suggested algorithm to obtain the best estimate for the woman is, in order of priority: (1) ultrasound at <24 weeks by measuring, preferably, the crown–rump length (8–14 weeks) or head circumference (15–24 weeks); (2) if ultrasound not available, validated newborn gestational age assessment; (3) if (1) and (2) not available, the date of the last menstrual period or the symphysis–fundal height
•Analysis using birthweight or low birthweight should be controlled for gestational age
•Methods used to estimate gestational age should have regular (yearly) standardisation sessions and ongoing quality control (http://www.medscinet.net/intergrowth/protocol.aspx)
•Bias caused by the selection of a particular dating method—or no dating method—should always be considered as an alternative explanation for any identified associations^65^
**Confounders**
•Potential confounders should be diagnosed and included in the birthweight analysis

## Supporting information

The following supplementary materials are available for this article:

**Table S1.** Included studies.

**Table S2.** Reporting of weight measurements.
