# Supplementary material for: Malaria in pregnancy: the difficulties in measuring birthweight
Source: BJOG. 2011 Feb 18;118(6):671–8. doi: 10.1111/j.1471-0528.2010.02880.x (PMC3118281; doi:10.1111/j.1471-0528.2010.02880.x)
Supplement: Supplementary file 1 [file bjo0118-0671-SD1.doc]

**Table S1. Included studies**

| **Author** | **Study period** | **Continent** | **Trial Type** | **Malaria study type** |
| --- | --- | --- | --- | --- |
| Adam^13^ | 1998-2001 | Africa | Prospective non-comparative | Treatment |
| Bounyasong^14^ | 1995-1998 | Asia | RCT | Treatment |
| Browne^15^ | 1994-1995 | Africa | RCT | ITN |
| Challis^16^ | 2001-2002 | Africa | RCT | IPTp |
| Clerk^17^ | 2004 2007 | Africa | RCT | IPTp |
| Cot^18^,^19^ | 1991-1993 | Africa | RCT | Chemoprophylaxis |
| Cot^20^, ^21^ | 1987 1988 | Africa | RCT | Chemoprophylaxis |
| Deen^22^ | 1999 | Africa | Retrospective | Accidental exposure |
| Denoeud^23^ | 2004-2005 | Africa | Observational | Chemoprophylaxis |
| Dolan^24^ | 1990-1992 | Asia | RCT | ITN |
| Dorman^25, 26^ | 1996-1997 | Africa | Observational within RCT | IPTp |
| Egwunyenga^27^ | 1992 | Africa | Observational | Chemoprophylaxis |
| Ekejindu^28^ | NA | Africa | Cross sectional | Not reported |
| Falade^29, 30^ | 2003-2004 | Africa | Observational | IPTp |
| Filler^31^ | 2002-2005 | Africa | RCT non blinded | IPTp |
| Fleming^32^ | 1980 | Africa | RCT | Chemoprophylaxis |
| Gies^33, 34^ | 2004-2006 | Africa | Observational | IPTp |
| Greenwood^35-37^ | 1984-1987 | Africa | RCT | Chemoprophylaxis |
| Hamer^38, 39^ | 2003-2004 | Africa | RCT | IPTp |
| Hamilton^40^ | 1965 | Africa | RCT | Chemoprophylaxis |
| Kalanda^41-44^ | 1993-1994 | Africa | Cross sectional | Non interventional |
| Kayentao^45^ | 1998-2001 | Africa | RCT | IPTp + Chemoprophylaxis |
| Larocque^46^ | 2003-2004 | S-America | RCT | Treatment |
| Mbaye^47^ | 2002-2004 | Africa | RCT | IPTp |
| Mbonye^48^,^49^ | NA | Africa | Community non RCT | IPTp |
| McGready^50^ | 2001-2003 | Asia | RCT | Treatment |
| Menendez^51^ | 1987-1990 | Africa | RCT | Chemoprophylaxis |
| Menendez^52, 53^ | 1987-1990 | Africa | RCT | Treatment |
| Menendez^54^ | 2003-2005 | Africa | RCT | IPTp + ITN |
| Msyamboza^55^ | 2002-2004 | Africa | Community non RCT | IPTp |
| Mutabingwa^56, 57^ | 1988-1991 | Africa | RCT | Chemoprophylaxis |
| Ndyomugyenyi^58^ | 1996-1998 | Africa | RCT | Chemoprophylaxis |
| Ndyomugyenyi^59^ | 1997-1998 | Africa | Retrospective | Labour record review |
| Nosten^60^ | 1987 | Asia | RCT | Pharmacokinetic |
| Nosten^61^ | 1987-1990 | Asia | RCT | Chemoprophylaxis |
| Rahimy^62^ | 1994 | Africa | Prospective non-comparative | Non interventional |
| Shulman^63^ | 1992-1995 | Africa | RCT | ITN |
| Steketee^64, 65^ | 1987-1990 | Africa | RCT | Chemoprophylaxis |
| Taha^66^ | 1989-1990 | Africa | Nested case control | Non interventional |
| Ter Kuile^67, 68^ | 1992-1999 | Africa | RCT | ITN |
| Tukur^69^ | 2002 | Africa | RCT | IPTp |
| Villamor^70, 71^ | 2000-2002 | Africa | RCT | Treatment |
| Villegas^72^ | 1998-2000 | Asia | RCT | Chemoprophylaxis |

Abbreviations: BW birth weight; IPTp Intermittent Preventive Treatment in pregnancy; ITN Insecticide Treated Net; NA not available; RCT Randomised controlled trial; S South.
