# Supplementary material for: Malaria in pregnancy: the difficulties in measuring birthweight
Source: BJOG. 2011 Feb 18;118(6):671–8. doi: 10.1111/j.1471-0528.2010.02880.x (PMC3118281; doi:10.1111/j.1471-0528.2010.02880.x)
Supplement: Supplementary file 2 [file bjo0118-0671-SD2.doc]

|  | **Author** | **Type scale** | **Reported precision scale (g)** | **Quotations of BW**^ | **Included for BW** | | | **Day of weight** | **Who is included** |
| --- | --- | --- | --- | --- | --- | --- | --- | --- | --- |
|  |  |  |  |  | **BW** | **Total** | **%** |  |  |
|  |  |  |  |  |  |  |  |  |  |
| **Weight scales** | Falade^29^ | Digital | 1 | 3204.3±487.2 (1500 – 4700) | 980 | 983 | 99 | NA | NA |
|  | Kalanda^41, 42, 44^ | Salter | 10 | **2843±533** | 1344 | 4104 | 33 | NA | Live singleton |
|  | Menendez ^54^ | Digital | 1 | 3003.55±522.69 | 990 | 1030 | 96 | NA | All |
|  | Mutabingwa^56^ | Hanging + Digital | 10-100 | 2.79±0.42 kg | 291 | 423 | 69 | NA | Term singleton |
|  | Ndyomugyenyi^59^ | NA | 100 | **2.930±0.54 kg** | 5991 | 5991 | 100 | NA | All** |
|  | Tukur^69^ | NA | 50 | 3.12±0.51 kg | 351 | 500 | 70 | NA | NA |
|  |  |  |  |  |  |  |  |  |  |
| **Scales + Timing** | Deen^22^ | Seca 835 | 10 | **2.87 [% CI 2.77-2.961]** | 195 | 459 | 42 | < 9 | All |
|  | Dolan^24^ | Salter | 10 | 2476±550 | 301 | 341 | 88 | < 1 | Live singleton |
|  | Dorman^25, 26^ | Digital | 5 | **LBW Y/N** | 340 | 854 | 40 | Birth | Singleton |
|  | Filler^31^ | Digital | 1 | 2.74±0.56 kg | 491 | 698 | 71 | < 1 | Live singleton |
|  | Gies^33, 34^ | UNICEF hanging | 25 | **2563 [% CI 2420–2706]** | 1281 | 1883 | 68 | < 1 + F | Singleton |
|  | Kayentao^45^ | Digital | NA | **2676±435** | 1062 | 1163 | 91 | < 1 | Live singleton |
|  | Larocque^46^ | Seca 334 + 345$ | 10 | 3104±441.54 | 950 | 1042 | 91 | < 2 | Live singleton |
|  | Mbonye^49^ | Salter | 50 | **3220 g [range 3186—3264]** | 1227 | 2785 | 44 | < 5 | Term singleton |
|  | Msyamboza ^55^ | Chasmor hanging~ | 100 | LBW Y/N | 1733 | 1752 | 99 | < 7 +F | Singleton |
|  | Ndyomugyenyi^58^ | Spring | 10-25 | 3007±4 | 472 | 860 | 55 | < 7 | Live singleton |
|  | Steketee^64, 65^ | Mettlerº | 1 | **2905 ± 461** | 1642 | 1835 | 89 | < 1 | Live singleton |
|  | ter Kuile^67^ | Salter 235,10S @ | 10 | 3.19 ± 0.02 kg | 833 | 833 | 100 | < 4 + F | Live singleton |
|  | Villamor^70^ | Beam balance | 10 | **LBW Y/N** | 258 | 275 | 94 | Birth | Live born |
|  | Villegas^72^ | Salter# | 50 | 2777 ± 435 (1650–4200) | 733 | 1000 | 73 | <3 | Live singleton |
|  |  |  |  |  |  |  |  |  |  |
| **Timing** | Bounyasong^14^ | NA | NA | **2785.00** | 57 | 60 | 95 | < 1 | NA |
|  | Browne^15^ | NA | NA | 2.19 [95CI 2.05-2.33] kg | 847 | 1961 | 43 | < 7 + F | All** |
|  | Challis^16^ | NA | NA | **3077±533** | 403 | 600 | 67 | < 1 | All |
|  | Clerk^17^ | NA | NA | 2790±452 | 1133 | 3643 | 31 | Birth + F | Singleton |
|  | Cot^18, 19^ | NA | NA | **3069.8±56.8** | 122 | 266 | 46 | Birth | Live singleton |
|  | Cot^20, 21^ | NA | NA | 2937.8±452.5 | 1148 | 1464 | 78 | Birth | Live singleton |
|  | Fleming^32^ | NA | NA | 2723 | 89 | 200 | 45 | Birth | All |
|  | Denoeud^23^ | NA | NA | 2850 (range: 800–5000) | 1087 | 1176 | 92 | Birth | Live singleton |
|  | Greenwood^35^ | NA | NA | **2790±400** | 730 | 1049 | 70 | <7 + F | Singleton |
|  | Mbaye^47^ | NA | NA | 3090 | 2002 | 2688 | 74 | <7 | NA |
|  | McGready^50^ | NA | NA | 2930 ± 648 (1000–4100) | 53 | 81 | 65 | <1 | Live singleton |
|  | Menendez^51^ | NA | NA | **3028 ± 414** | 182 | 230 | 79 | <1+F | Live singleton |
|  | Menendez^52, 53^ | NA | NA | 3103 | 450 | 550 | 82 | <7+F | Live singleton |
|  | Rahimy^62^ | NA | NA | **2500 ± 540** | 89 | 108 | 82 | Birth | Live singleton |
|  | Shulman^63^ | NA | NA | 2.8 (SE 0.06) kg | 130 | 503 | 26 | Birth | Live singleton |
|  | Taha^66^ | NA | NA | LBW Y/N | 2683 | 2683 | 100 | Birth | Live singleton |
|  |  |  |  |  |  |  |  |  |  |
| **None** | Adam^13^ | NA | NA | 3.45 ± 0.76 kg | 12 | 40 | 30 | NA | Live singleton |
|  | Egwunyenga^27^ | NA | NA | **2650 ± 240** | 284 | 656 | 43 | NA | Live born |
|  | Ekejindu^28^ | NA | NA | NA* | NA | 108 | NA | NA | NA |
|  | Hamer^38, 39^ | NA | NA | 2987 **±** 40.77 | 387 | 456 | 85 | NA | Live singleton |
|  | Hamilton^40^ | NA | NA | **3020 ± 597** | 1137 | 1846 | 62 | NA | NA |
|  | Nosten^60^ | NA | NA | 3.4 **±** 0.5 kg | 20 | 20 | 100 | NA | Live singleton |
|  | Nosten^61^ | NA | NA | **2877 ± 433** | 311 | 339 | 92 | NA | NA |

**Table S2 Reporting of weight measurements**

Abbreviations: F Formula to calculate BW if day of weight not within 1 day; NA not available

^ Bold if significant effect on BW was reported

* There was no report of BW, but IUGR was reported in results section and LBW in abstract^28^

** Unclear whether twins and stillborns were included for birth weight analysis^15, 59^

$ Seca corp., Baltimore, MD, USA

~ Chasmor, London, UK

º Mettler, Rite weight, Inc, Duluth, GA, USA
@ Salter, Smethwick, UK

# Salter, Birmingham UK
